# Evaluating the Potential Fitness Effects of Chinook Salmon (*Oncorhynchus tshawytscha*) Aquaculture Using Non-Invasive Population Genomic Analyses of MHC Nucleotide Substitution Spectra

**DOI:** 10.3390/ani13040593

**Published:** 2023-02-08

**Authors:** Evan J. Wilson, Andrew M. Shedlock

**Affiliations:** 1 Department of Biology, College of Charleston, Charleston, SC 29424, USA; 2 College of Graduate Studies, Medical University of South Carolina, Charleston, SC 29425, USA; 3 Hollings Marine Laboratory, Marine Genomics Group, Charleston, SC 29412, USA

**Keywords:** *Oncorhynchus*, Chinook salmon, Alaska, Susitna River, MHC Class I and II loci, population genomics, heterozygosity, selection, non-synonymous substitution, peptide binding region

## Abstract

**Simple Summary:**

DNA sequence variation in the major histocompatibility complex (MHC) is vital to the health of the vertebrate adaptive immune system. Consequently, MHC Class I and II loci have evolved to become the most polymorphic vertebrate genes known. This extraordinary level of variation makes MHC immunity genes both prime indicators of individual fitness as well as highly sensitive biomarkers for assessing the population genetic health of wildlife, and by extension, sustainable management of biodiversity. We examined levels of MHC locus-specific DNA haplotype variation in both wild and hatchery-reared Chinook salmon (*Oncorhynchus tshawytscha*) sampled from the Susitna River Basin where wild Alaskan Chinook stocks are declining regionally and creating incentives for expanded aquaculture. Our non-invasive population analyses indicated higher levels of observed heterozygosity than expected heterozygosity at the Class I and II loci and minimal genetic differentiation between spatially distinct subpopulations. Class I sequences also showed evidence of balancing selection, despite high rates of non-synonymous substitutions observed, specifically at the peptide binding regions of both MHC genes. Taken together, these results suggest that current management practices for Chinook populations in the Susitna Basin are not likely causing any detrimental biological changes in salmon in relation to immune health. Moreover, the immune health of the hatchery fish, at least in the context of the two populations studied, matches that of the wild fish. Our locus-specific work invites a genome-wide survey such as mapping Single Nucleotide Polymorphisms to better understand the role of the MHC in optimal mate choice and to establish MHC relatedness as a proxy for maintaining genetic variability throughout the entire genomes of fish within populations.

**Abstract:**

Genetic diversity plays a vital role in the adaptability of salmon to changing environmental conditions that can introduce new selective pressures on populations. Variability among local subpopulations may increase the chance that certain advantageous genes are passed down to future generations to mitigate susceptibility to novel diseases, warming oceans, loss of genetic stocks, and ocean acidification. Class I and II genes of the major histocompatibility complex (MHC) are crucial for the fitness of Chinook salmon due to the role they play in disease and pathogen resistance. The objective of this study was to assess the DNA sequence variability among wild and hatchery populations of Alaskan Chinook salmon at the class I α1 and class II β1 exons of the MHC. We hypothesized that the 96 wild samples taken from the Deshka River would display greater levels of observed heterozygosity (Ho) relative to expected heterozygosity (He) in suggesting that individuals with similar phenotypes mate with one another more frequently than would be expected under random mating patterns. Conversely, since no mate selection occurs in the William Jack Hernandez Sport Fish hatchery, we would not expect to see this discrepancy (He = Ho) in the 96 hatchery fish tested in this study. Alternatively, we hypothesized that post-mating selection is driving higher levels of observed heterozygosity as opposed to mate selection. If this is the case, we will observe higher than expected levels of heterozygosity among hatchery salmon. Both populations displayed higher levels of observed heterozygosity than expected heterozygosity at the Class I and II loci but genetic differentiation between the spatially distinct communities was minimal. Class I sequences showed evidence of balancing selection, despite high rates of non-synonymous substitutions observed, specifically at the peptide binding regions of both MHC genes.

## 1. Introduction

### 1.1. Scope and Aims of This Study

In this study, we examine the genetic variation at the major histocompatibility complex (MHC) in two populations of Chinook salmon (*Oncorhynchus tshawytscha*); one occurring in the Deshka River, Alaska, and the other derived from the William Jack Hernandez Sport Fish Hatchery in Anchorage. The prevalence of Chinook salmon declined over the past 50 years [1], and the acknowledgment of locally adapted functional loci, such as the MHC, may be important when population conservation and revitalization plans for Chinook salmon are developed [2]. The present research expands upon earlier studies [1,2,3,4] by examining the genetic variation observed within a wild population (Deshka River) and a hatchery population (Hernandez Sport Fish Hatchery) of Chinook salmon at the MHC class I-α1 and class II-β1 loci. Genetic polymorphisms are quantified using target-primed polymerase chain reaction (PCR) amplification followed by single stranded Sanger di-deoxy chain termination fluorescent four-color chemistry analyzed on an Applied Biosystems capillary array DNA sequencer (Eurofins Genomics, LLC, Louisville, KY, USA). We also assess selection at the DNA level by examining patterns of MHC sequence substitutions to determine whether haplotypes from each population show evidence for balancing or directional selection. Our findings from this study further the understanding of evolution at both MHC loci examined and provide novel insights on the locus- and site-specific distributions of heritable immunogenetic polymorphisms of the Deshka River and William Jack Hernandez Chinook salmon populations.

### 1.2. Potential Impact of Hatchery Releases

Salmon consumption has increased three-fold since the 1980s [5]. The added pressure of increasing demand has required human intervention to maintain salmon populations. As a result, salmon aquaculture is considered one of the fastest growing food production fields in the world, with 70% of the current salmon market being driven by hatchery-reared salmon [5]. The impact of the expanded aquaculture of salmon has been a hot topic in recent years. One of the concerns with increasing hatchery output is the potential for the introgression of genes from hatchery fish into native progenies and the potential loss of genetic diversity among native populations. These forms of release can cause genetic change by means of changes in genetic variability, loss of adaptation, change in population composition, and change in population structure [6]. Population genetics theory posits that genetic diversity is significantly reduced only in small populations; however, this view of population genetics only holds true in regard to genetically effective populations (Ne), which may be several orders of magnitude lower than the overall census population size [7]. The short-term effective population size of marine species may be represented by only hundreds or thousands of individuals depending on reproductive success, and the variation in year-to-year stock strength of serial spawners, as is the case with many salmon species [7]. A modern-day example of this can be observed in the endangered winter-run Chinook salmon of the Sacramento Valley. As a result of supplementation breeding to support the endangered population, a recent phylogenetic analysis indicated that the most commonly observed locus among winter-run Chinook, Onts-wr1 is not found in late fall and is only found in low frequencies among spring and fall runs [8,9]. The winter-run was determined to be quite distinct from the other runs and homogeneous over different years. This suggests that effects due to limited population size have reduced the amount of synonymous variation [8,9].

Certain domesticated species of salmon have been shown to exhibit loss of heterozygosity and allelic diversity leading to a potentially lower adaptive resiliency among cultured populations, resulting in reduced viability in wild environments [10]. These genetic weaknesses are believed to be the result of changes in the environment that the fish are subjected to, as well as selective breeding that is primarily focused on producing fish of greater size. In a similar study, it was revealed that first-generation hatchery fish had nearly double the lifetime reproductive success of wild fish under identical conditions, and that individuals who were best adapted to a captive environment produced offspring that performed the worst in the wild between hatchery and wild specimens [10,11].

Assessing MHC diversity within other salmonid species that are more abundantly used in aquaculture may also offer insight into the potential for extensive and expansive exposure and infection from diseases and parasites. Recent studies have revealed that long-distance transmission of novel diseases such as piscine reovirus (PRV), and heart skeletal and muscle inflammation (HSMI) is believed to be largely driven by the aquaculture industry, as it plays the biggest role in the transport of this virus due to its congregation in larger groupings and at higher densities than wild salmon populations [12]. Atlantic salmon have experienced significant genetic bottlenecking on a global scale likely due to their role as key species within global aquaculture. Recent studies showed that there tended to be a deficiency of homozygous individuals in the populations that had been exposed to and survived the M74 syndrome in Norwegian populations [13,14]. This indicates a tendency for selection against homozygotes during exposure to the syndrome, and that natural selection may favor heterozygous individuals [13,14].

## 2. Materials and Methods

### 2.1. DNA Purification, PCR Amplification, and DNA Sequencing

**Figure 1:**
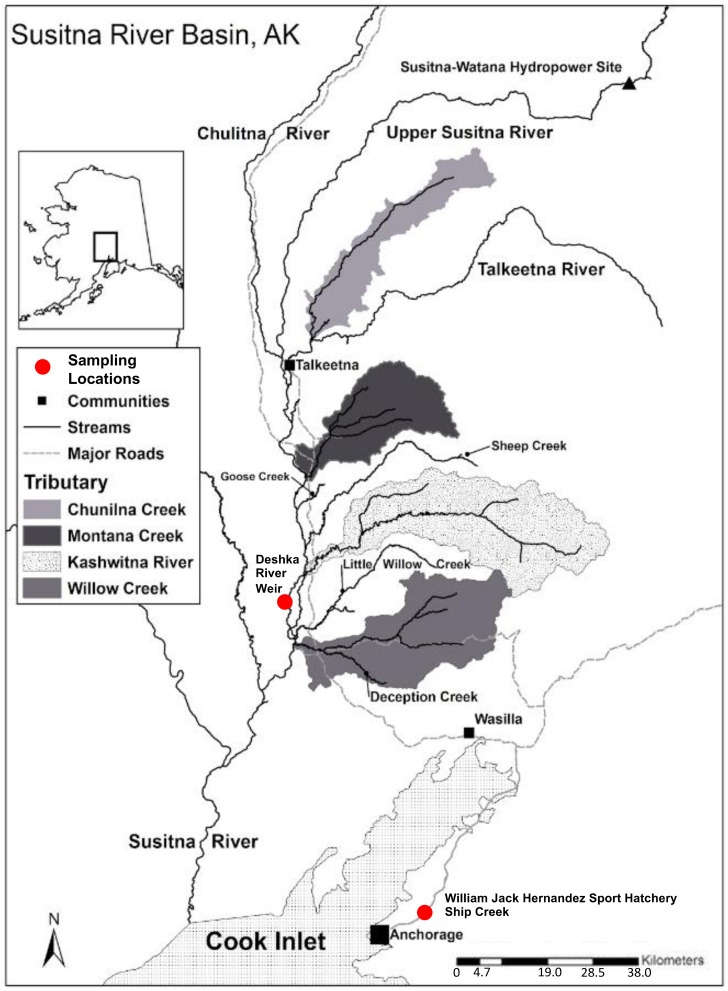
Map of Susitna River Basin and sampling locations for both wild and hatchery Chinook salmon. GIS data taken from University of Alaska, Fairbanks, Cooperative Fish and Wildlife Research Unit [16].

Genomic DNA was extracted from axillary fin clips sampled from 96 wild individuals and 96 hatchery-derived Chinook salmon from the Deshka River Weir and the William Jack Hernandez Hatchery in Southcentral Alaska (Figure 1). Isolation of genomic DNA from 95% ethanol-preserved fin clips was performed with Qiagen Blood and Tissue DNA Extraction Kits (Qiagen Sciences Inc., Germantown, MD, USA) via digestion with proteinase K (20 mg/mL); DNA elution with 100 uL of AE elution buffer. Fin clips from the dorsal region were collected from late spring/early summer run adult and jack Chinook salmon from the same localities as above and preserved in 95% ethanol for storage and transportation prior to DNA purification using the same methods described above for axillary fin clips but with extended proteinase K lysis as described in [4]. Isolation of genomic DNA from an ancillary Chinook scale sample collected from Ship Creek, Anchorage, AK (Figure 1) was performed following the Qiagen Blood and Tissue DNA Extraction Kits via extended digestion with proteinase K (40 mg/mL) for 3 nights. DNA was then eluted with 100 uL of AE buffer.

PCR primer sequences for each MHC locus (class I α1 and class II β1) are summarized in Table 1 following the primer design protocols described in [4,15]. Initial PCR optimization experiments were conducted to test each primer using 4 uL of DNA sample. PCR products were electrophoresed on an agarose (1.3% *w*/*v*) gel to visualize target-locus amplification quality and controls for contamination. Examples of successful PCR reactions are indicated in Supplementary Figure S1 by distinct banding at the 213 and 228 bp sequence length ladder shown on the left. MHC PCR products were purified using a Qiaquick PCR cleanup kit (Qiagen Sciences Inc., Germantown, MD, USA) and sequenced with locus-specific priming via Sanger di-deoxy chain termination fluorescent four-color chemistry on an Applied Biosystems capillary array DNA sequencer (Eurofins Genomics, LLC, Louisville, KY, USA).

### 2.2. Population Genetic Analyses

Population genetic tests for Hardy–Weinberg equilibrium, linkage disequilibrium, Analysis of Molecular Variance (AMOVA), average heterozygosity, and F-statistics were carried out using standard population analysis software applications bundled in GenAlEx v.6.5 [17] and ARLEQUIN v. 3.5.2 [18]. Tajima’s D statistic was measured using MEGA X [19]. This analysis involved 205 amino acid sequences. The coding data were translated assuming a genetic code table. All ambiguous positions were removed for each sequence pair (pairwise deletion option). Individual sequence reads were aligned utilizing the CLUSTAL-codon V algorithm bundled in the MEGA X software package [19] for each of four mapped MHC exonic regions captured by the two sets of primers summarized in Table 1. Haplotype sequence variation in MHC loci was analyzed for evidence of reduced polymorphism in nucleotide substitution spectra using the molecular evolutionary analysis programs in MEGA X. The results of the present Chinook salmon study were compared against Chinook salmon and other closely related salmonid species for the same MHC class I and II loci accessioned in NCBI/Genbank. The MHC primer sequences are designed to amplify the exons encoding the peptide binding region located on the α1 chain of the MHC class I and the β1 chain of the MHC class II. Sequences of cleaned PCR products were run through the BLAST program for annotation confirmation against the NCBI publicly available genomic database prior to being entered into the MEGA X program and aligned using the “MUSCLE” exonic sequence alignment toolkit. From these alignments, anchored phylogenetic trees were developed for MHC class I and II genes utilizing the Atlantic salmon as the anchored outgroup. Neighbor-joining trees were constructed using p-distance including all the amino acids, with bootstrap values obtained after 1000 iterations. Independent assessment of NJ tree results was tested using Maximum Parsimony methods of phylogenetic inference and bootstrapping.

STRUCTURE v 2.3.4 [20] was used to infer genetically distinct groups among the two populations. The program was run using the admixture model utilizing a burn-in length period of 10,000 iterations for 10,000 Markov chain Monte Carlo (MCMC) repetitions and testing for K (number of populations) between 2 and 7 for 10 simulations. The creation of minimum spanning and transitive consistency score (TCS) haplotype networks was conducted using the POPART (v. 1.7) software [21,22,23]. The TCS methodology uses nucleotide sequence data to infer population level genealogical networks, even in instances where divergence is low by collapsing closely related sequences into haplotypes allowing the program to estimate frequencies of haplotypes and probable outgroups [21]. A minimum spanning tree connects all present haplotypes without further inference of ancestral nodes, so that total length of branches is kept minimal [22].

### 2.3. Assessment of Selection from Nucleotide Substitution Spectra

We used the Datamonkey HyPhy [24,25,26] programs to calculate ratios of non-synonymous to synonymous substitutions (dN/dS) to assess the level and type of site- and locus-specific selection operating across aligned haplotypes. FUBAR (Fast Unconstrained Bayesian Approximation) and SLAC (Single-Likelihood Ancestor Counting) methods measured sites of positive and negative selection among MHC nucleotide sequences. The SLAC methodology involves counting non-synonymous and synonymous changes and testing whether site-specific non-synonymous changes (dN) differ from the number of synonymous changes per synonymous site (dS) [25]. The FUBAR (mean posterior substitution rates) methodology uses a Markov chain Monte Carlo (MCMC) simulation that averages a large number of predefined site classes in an unconstrained manner, allowing sites influenced by positive and purifying selection to be identified [24]. The MEME methodology uses empirical sequence data from a variety of taxonomic backgrounds in unison with comprehensive phylogenetic models to identify instances of site-specific episodic and pervasive positive selection [26].

## 3. Results

### 3.1. Sequence Variation

A total of 270 aligned original protein coding sequences for 126 PCR products of the MHC class I-α1 locus and 144 PCR products of the MHC class II-β1 locus sampled from 192 individuals were deposited into Genbank (see Data Availability Statement below for Accession Nos.) Among all individuals, there were 34 alleles found among the MHC Class I A1 loci (Supplementary Table S1) and 34 present within the MHC Class II B1 locus (Supplementary Table S2). The mean pairwise diversity of nucleotide sequences at the A1 loci was 95%, whereas the pairwise amino acid similarity at the B1 loci was 98%.

There was considerable differentiation in the overall variance components present in the two loci due to the discrepancies in alleles present among each class, as well as overall sequence variation (34 Class I and 4 Class II, Table 2).

### 3.2. Heterozygosity

The levels of observed heterozygosity exceeded the levels of expected heterozygosity for both hatchery (Ho = 0.898; He = 0.478, Ho = observed heterozygosity; He = expected heterozygosity) and wild (Ho = 0.879; He = 0.468) populations for the Class I loci as shown in Table 3. Observed exceeded expected heterozygosity at the Class II loci (Ho = 0.817; He = 0.409 for both populations), but the fixation index value was 0.0001, signifying no genetic subdivision between populations (Table 4). The total observed heterozygosity (including NCBI Chinook, Pink salmon, Atlantic salmon, and Rainbow trout) also exceeded the expected levels of expected heterozygosity (Ho = 0.838; He = 0.450) for the 205 samples included in the Class I calculations. There were 38 loci among hatchery sequences that were not in the Hardy–Weinberg Equilibrium (HWE), which assumes no mutation, no gene flow, a large population size, random mating, and no natural selection is present among sequences. Wild sequences had 41 loci that rejected HWE and there were 147 total loci that deviated from the HWE among all sequences. Mean nucleotide diversity across all populations was 0.06, 0.037 for hatchery sequences, and 0.046 for wild sequences. Only 11 loci deviated from the HWE for the Class II loci among 145 sequences. The total observed heterozygosity for this locus was 0.814 but only included an additional two consensus sequences from NCBI [2,4]. The Shannon index is a measure of diversity of a species in a community, where a higher index value indicates higher diversity. The Shannon index for the hatchery sequences was 0.569, and it was 0.567 for wild sequences.

### 3.3. Rates of Substitution

**Figure 2:**
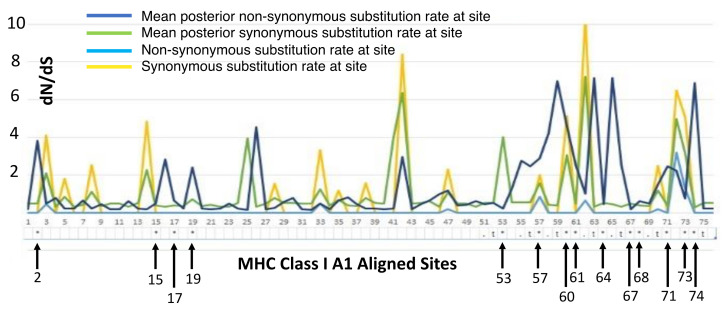
MEME overlay plot for codons associated with the mammalian peptide binding region (PBR) are shown numerically below the *X*-axis with notations for the location of amino acids associated with the PBR for the A1 exon following [27].

**Figure 3:**
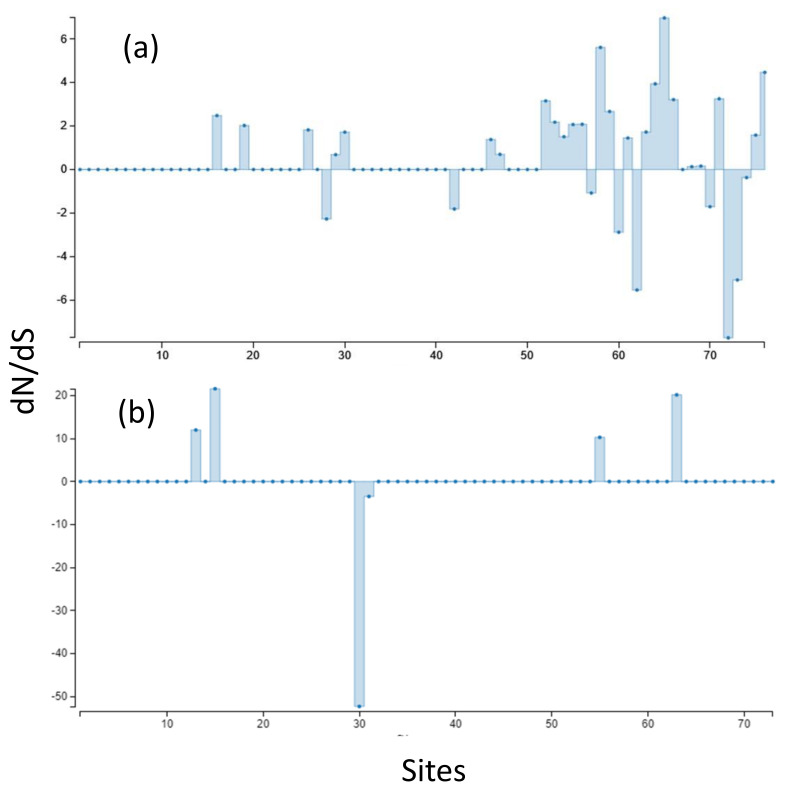
(**a**) SLAC-inferred dN/dS ratios, scaled by length of tested branches for MHC Class I A1. (**b**) MHC Class II B1. Synonymous substitutions are bars above 0 (zero indicates no selection or neutrality) and non-synonymous substitutions are indicated by bars below 0.

**Figure 4:**
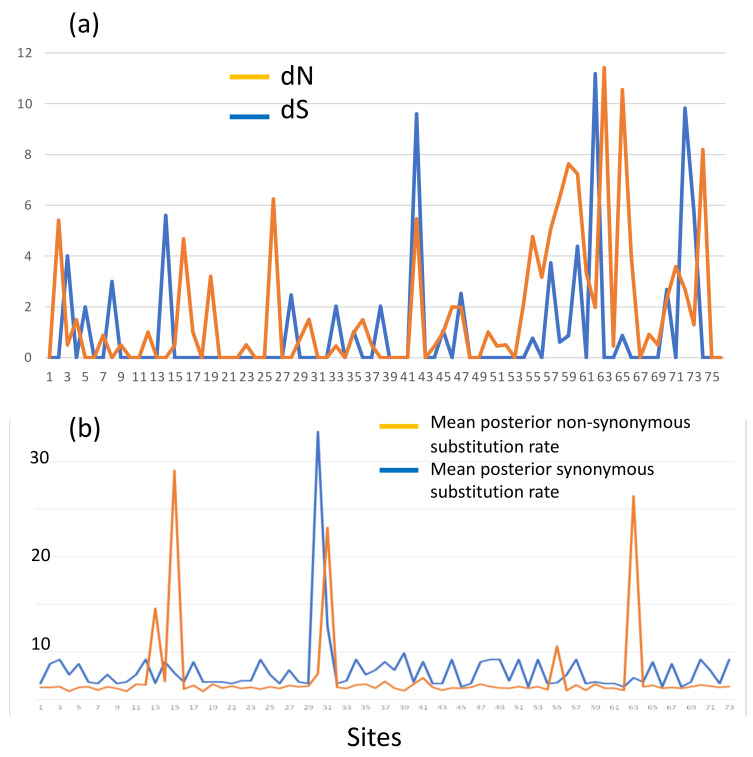
FUBAR: Non-synonymous substitutions for (**a**) Class I A1 loci, dN indicated in orange, dS in blue. (**b**) Class II B1 loci. The *Y*-axis shows substitutions per site, the *X*-axis indicates the codon site.

Aligned sequences were examined for evolutionary signatures present within sequence data using a multimethod approach with programs bundled in the software packages Single-Likelihood Ancestor Counting (SLAC), Fast Unconstrained Bayesian Approximation (FUBAR), and the Mixed Effect Model of Evolution (MEME). These programs were implemented as grounds for identifying pervasive diversifying or purifying selection (SLAC and FUBAR), and episodic diversifying selection (MEME) present within wild and hatchery sequences. Each methodology analyzed 142 sequences at 76 sites (Class I A1) and 145 sequences at 73 sites (Class II B1) for the two MHC genes under investigation. The MEME methodology (Figure 2) revealed episodic positive selection at 14 sites among A1 sequences and none for the B1 loci. The SLAC methodology found evidence of positive/diversifying selection at nine sites and negative/purifying selection at six sites (*p* < 0.1) among A1 sequences (Figure 3). The SLAC method showed no sites under selective pressure for the B1 loci, but sites under selection are observable when analyzing the graphical output of the data (Figure 3). There appears to be four sites under positive selection, with one site under heavy negative selection. The FUBAR methodology highlighted 14 sites of positive and 6 sites of negative selection (Figure 4) present among A1 MHC sequences, with 2 sites under positive selection (15 and 63) and 1 site under negative selection (site 31) at the B1 loci (Figure 4).

Rates of substitution were much higher at putative peptide binding regions than in non-binding codons (Table 5). PBR codons had a dN/dS of 1.7 suggesting evidence of positive selection, whereas non-PBR codons were more neutral (1.048) in the Class II loci. The overall dN/dS rate of the Class II loci was 1.115, suggesting the presence of balancing selection. The Class I loci showed even higher rates of non-synonymous substitutions in the codons of the PBR (dN/dS = 2.94), suggesting even stronger positive selective pressures. The non-PBR sites were much more homogenious, and total dN/dS for the loci among all individuals was 1.73.

### 3.4. Population Structure

**Figure 5:**
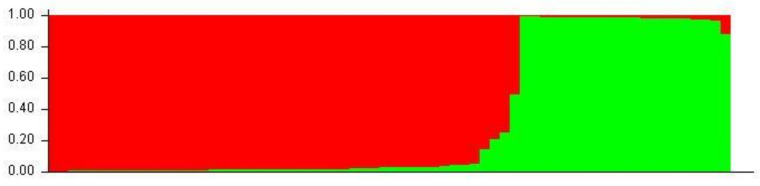
A1 STRUCTURE plot (two populations). The *Y*-axis of the STRUCTURE plot serves as an indicator of percentage derived from lineage. The *X*-axis indicates individuals sampled among populations. Red clustering shows proportions of parentage derived from lineage 1 and green clustering shows proportions derived from lineage 2.

**Figure 6:**
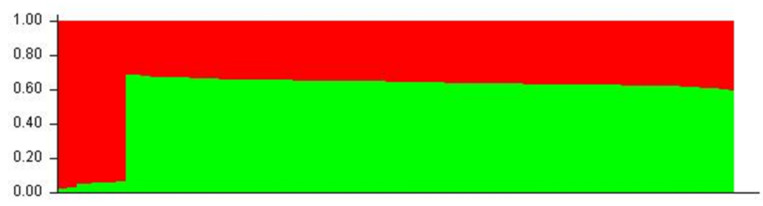
B1 STRUCTURE plot (two populations). Red clustering shows proportions of individuals from lineage 1; green clustering shows proportions of individuals from lineage 2.

Aligned sequences were analyzed using the STRUCTURE (v. 2.3.4) software to assess inferred ancestry of individuals for both the MHC Class I and II loci. STRUCTURE identifies subpopulations of samples by detecting differences in allele frequencies among samples. Figure 5 shows the Class I A1 simulation where 71% of individuals are clustered into lineage 1 and the remaining are from lineage 2. All but four of the individuals in lineage 2 were from the wild or hatchery populations. Those that are not were wild Chinook accessioned from NCBI. In the Class II B1 simulation (Figure 6), parameters were designed to assess the 138 samples at 213 loci within 2 populations. The simulation was run for 10,000 replications with a 10,000 burn-in period and returned two inferred population clusters (cluster 1~58.6%; cluster 2~41.4%). The vast majority of samples were predominately composed of the cluster 1 lineage (60–70%) with the remaining ancestry being derived from cluster 2 (30–40%); However, 14 individuals (HB7, HC7, HC9, HD9, HE4, HE7, HF3, HF8, HG4, HH11, HH5, WA8, WA9, and WE3) showed considerable differentiation in ancestry, with >90% of ancestry being derived from cluster 2. Additional Chinook sequences from British Columbia were included to highlight population structure differentiation interspecifically. Other salmonid species such as *Salmo salar* (Atlantic salmon); *Oncorhynchus gorbuscha* (Pink salmon); and *Oncorhynchus mykss* (Rainbow trout) were also added to display haplotypic clustering and genetic variation among populations.

**Figure 7:**
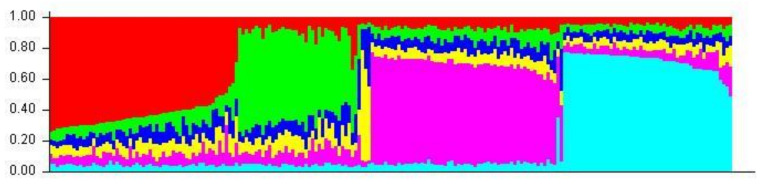
A1 STRUCTURE plot (six populations)—included in this STRUCTURE bar plot are accessioned sequences from published literature found on NCBI/Genbank [2,4].

Distinct clustering can be seen in the STRUCTURE plot assuming six populations (Figure 7) where homologous individuals are indicated by blocks of predominantly red-coded bars; allele-diverse individuals are indicated by predominantly green-coded bars; closely related salmonids (*Salmo salar*; *Oncorhynchus mykiss*; *Oncorhynchus gorbuscha*) are indicated by predominantly yellow-coded bars; and British Columbian lineages are indicated by predominantly purple or light blue-coded bars [2,4].

**Figure 8:**
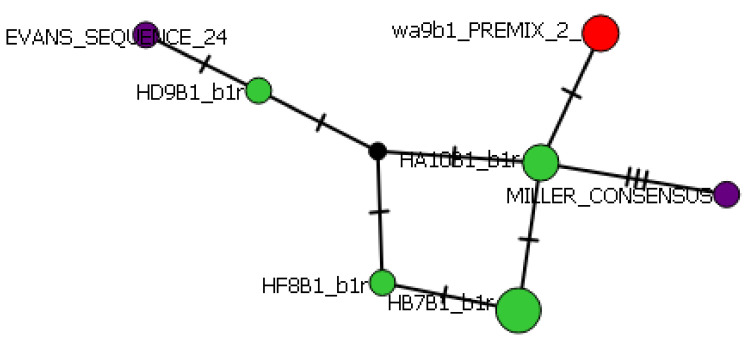
Class II haplotype network. Hatchery alleles are indicated by green dots, wild by red, and consensus NCBI sequences by purple.

Using the median-joining network method, phylogenetic maps created in PopART (version 1.7) show the distances of each allele, and their differentiation from their most closely related individual (indicated by hash lines on each connecting arm). PopART allows for visualization of intraspecific relationships and haplotype inference. The central web of alleles is only differentiated from two of the connecting arms by one variation in the amino acid sequence but show considerable differentiation from the Evans and Miller consensus sequences (derived from British Columbian Chinook salmon) [2,4]. These mutations are shown as a hatch marks on the connecting arms. The Class II haplotype network shows the most variation between the Miller sequence and the additional seven alleles present (Figure 8).

**Figure 9:**
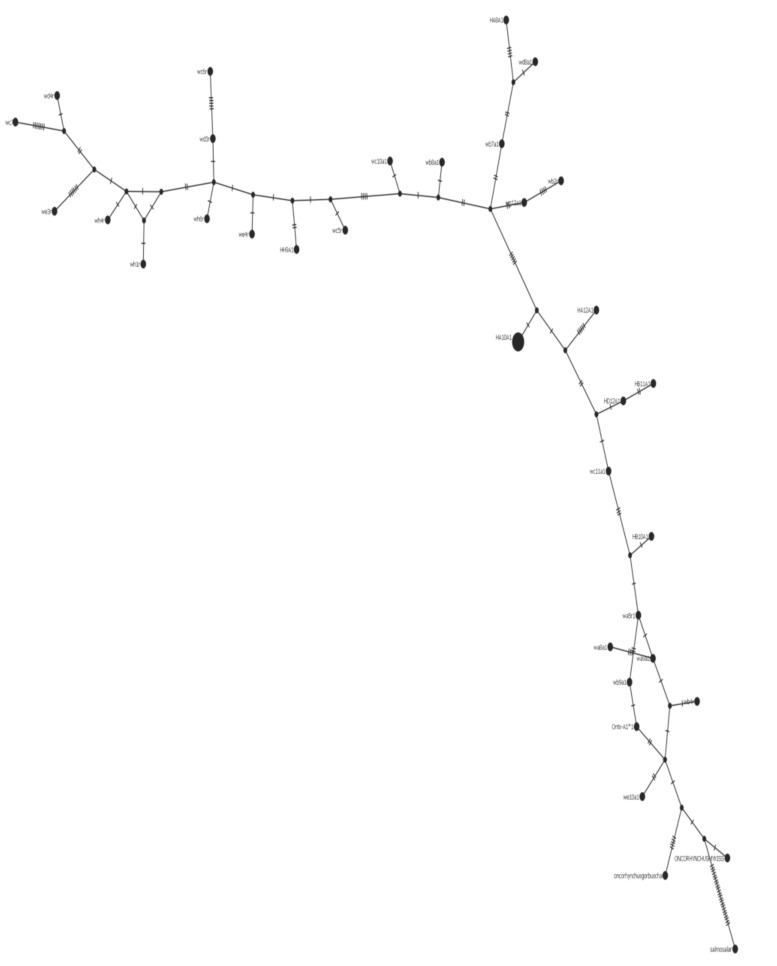
Class I haplotype network. The lower right branch is made up of the *Salmo salar*, *Oncorhynchus mykiss*, *Oncorhynchus gorbuscha*, and closely related wild samples. The center of the branch includes predominantly hatchery samples.

Using the TCS network method, the Class I haplotype network shows a more substantial amount of variation. The central web (Figure 9, bottom right) shows the close relationship between the various salmonid species (*Oncorhynchus mykiss*; *Oncorhynchus gorbuscha*; *Salmo salar*; Miller sequences (Onts-A1*1)) [2,4] included in this haplotype network. There are three main branches within this joining network, with a fourth suggesting radiation from the Atlantic salmon as it is the last common ancestor of the *Oncorhynchus* phylogeny. Nucleotide diversity among the sequences was 0.06, where 36 of the 56 segregating sites were identifiable as parsimony-informative sites.

## 4. Discussion

The main objective of this study was to test competing hypotheses determining the role of mate selection and inbreeding avoidance as drivers of increased heterozygosity among hatchery and wild Chinook salmon populations in Southcentral Alaska. It also serves as a comparative analysis of genetic variability retention among wild and hatchery populations, and as a measure of genetic and overall health. Additionally, it provides a comparative analysis of genetic variability retention among wild and hatchery populations. The findings of this study underscore the high level of genetic variability present among MHC Class I genes in both sample populations of Chinook salmon while also revealing lower-than-anticipated levels of polymorphism found in the MHC Class II gene that has been observed in previous studies of Chinook salmon [2]. Tests of non-synonymous and synonymous substitutions revealed complex signatures of site-specific selective pressures driving the molecular evolution of MHC genes in both hatchery and wild populations. Levels of non-synonymous substitution were considerably higher in PBRs (Table 5) than those of other MHC class I and II codons that exhibit signatures of directional positive selection. This suggests the potential for novel, advantageous adaptations throughout both populations that can be linked to predictive models of MHC three-dimensional protein structure and function. The high dN/dS ratio posits that selective pressures favor heterozygous individuals and is consistent with the literature pertaining to Chinook salmon MHC genes, salmonid species, and other teleost fish species [2,3,4,28]. Conversely, the Class II exon rates of dN/dS differ slightly from previous studies that suggest the presence of homogenizing selection across spatially different Chinook salmon populations [2,29].

This could mean that there are unique selective pressures between the two classes of MHC genes at the sequence level. This could also be a product of a comparatively smaller sample size (96 individuals for each population) than utilized in similar studies [2,3,4]. A study on guppies (*Poecilia reticulata*) by Fraser and Neff [30] stated that common pathogens across populations may be the driving force behind the lack of genetic divergence in relation to microsatellite loci observed at the Class II B gene. Furthermore, a study conducted on a closely related salmonid species (*Salmo trutta*) determined that individuals retaining rare alleles displayed better fitness regardless of zygosity, arguing that balancing selection was driven by a rare allelic advantage instead of heterozygote/divergent allelic advantage [31]. Ultimately, the highly polymorphic properties of MHC genes bode well for the adaptability necessary to interact with a plethora of pathogenic threats that are increasing with a changing climate and suggest that the amino acid sequences of the PBR feature selective pressures that favor heterozygous individuals.

The presence of particular alleles was generally not relegated to either population of Chinook salmon in this study as multiple alleles were present within both hatchery and wild individuals. All four alleles were found among hatchery fish at the Class II B1 locus and three of the four were present in the wild stock. In total, Twenty-seven of the 34 MHC Class I alleles were present within the wild stock and 14 were present among hatchery salmon. Other studies on salmonid species have shown higher rates of allelic diversity (albeit, with comparatively larger sample sizes). The Landry and Bernatchez [32] study of the Class II loci of Atlantic salmon reported 14 alleles present within 18 populations. Additionally, Miller et al. [3] identified 11 Class II alleles among 31 populations of Sockeye salmon and Evans et al. [2] reported 17 among the 365 Chinook salmon they sampled. Conversely, a previous study by Miller et al. [4] resulted in similar allelic frequencies to the present study and found only three Class II alleles present among two populations of 40 Chinook salmon. The Class I locus showed more variability between the two populations with hatchery and wild stocks possessing population-specific alleles. This is to be expected as they are spatially distinct despite their proximity to one another.

This pattern was also reflected by the statistical analyses of population structuring among the sample treatments. STRUCTURE (2.3.4) plots partitioned Class II genetic variants into subpopulations based on patterns of similarity. Of the 14 individuals denoted as being derived ancestrally from population 2, 11 were hatchery and 3 were wild. Thus, although there was inferred clustering based on genetic differentiation, it was not isolated to one population. Similar clustering was seen at the Class I locus. Of the 51 individuals clustered into population 2, 40 were wild stock and 11 were hatchery. The discrepancy here is likely a result of differentiation in the final number of individuals used for each calculation (86 wild vs. 48 hatchery) but shows that these alleles persist across geographically divergent lineages. The results of the PopART haplotype networks reiterates the presence of intermixed genetic ancestry among the sampled individuals. While there appears to be distinct haplotype clustering among the samples, the hatchery and wild subpopulations are not genotypically mutually exclusive.

Reviewing the results of the AMOVA conducted for the Class I and II loci (including the sequences from [2,4]), it can be determined that there is a lower overall level of variance within the sequences themselves but a greater percentage permeating interspecifically. AMOVA calculations for both MHC loci show considerably higher rates of variation within the distinct populations but there is still little in the way of variation between species of Pacific salmonids (generally < 5%). This suggests that much of the allelic variation observed within these sequences likely occurred during or sometime after the radiation of Pacific salmonid species [3,4]. The inclusion of sequences from NCBI likely results in some inflation of the interspecific variation, but also shows that ancestral lineages within Class I and II have not persisted throughout Pacific salmonids. The variance in the case of population genetics is increased by genetic drift, or the variance in relative frequency of genotypic differentiation present within a population. This includes the chance disappearance of certain alleles as a result of mortality or the lack of reproduction by carriers of rare alleles within a population. Variance among populations is lowered through gene flow (movement of genes into or out of a population) and genetic mutation.

Rates of genetic mutations are reflected in the various methodologies (SLAC, MEME, and FUBAR) utilized to measure non-synonymous and synonymous substitutions present within the sequences. The SLAC-methodology-calculated dN/dS ratios in Figure 3 display overall as well as site-specific dN/dS ratios, scaled by the length of the branches tested. At synonymous substitution sites, changes in the protein coding sequence do not result in changes in the amino acid sequence, these are known as a silent mutations [33]. Conversely, non-synonymous mutations result in changes in the amino acid sequence, resulting in biological change in the organism [33]. In terms of biological evolution, positive selection promotes the retention of beneficial alleles, whereas negative selection ensures deleterious mutations do not run rampant within a population. Purifying (negative) selection occurs either through eradication via non-random mating or through the process of genetic drift (change in the frequency of an existing allele in a population due to random sampling of organisms) [34]. Scaling selection coefficients help to distinguish purifying selection among synonymous substitution sites; if synonymous substitutions differ in added fitness, dN/dS ratios can result in arbitrarily high values even if purifying selection is the principle selective pressure [35]. The SLAC methodology involves counting non-synonymous and synonymous changes and testing whether site-specific non-synonymous changes (dN) differ from the number of synonymous changes per synonymous site (dS) [26]. dN/dS is assumed to evolve in a neutral manner (=1), whereas a dN/dS ratio >1 suggests positive selection, and a ratio <1 is under constraint. Figure 3 and Figure 4 show site-specific non-synonymous and synonymous substitutions independently for both MHC genes. As previously mentioned, SLAC coefficients identified nine sites of positive selection and six sites under negative selection at the A1 locus (assuming a *p* < 0.1), but there is evidence of additional sites (13 and 55) that may be under weaker selective pressures throughout the amino acid sequences (Figure 4). The FUBAR methodology highlighted 14 sites of positive and 6 sites of negative selection (Figure 4) present among A1 MHC sequences, with 2 sites under positive selection (15 and 63) and 1 site under negative selection (site 31) at the B1 locus (Figure 3). Finally, the MEME methodology reiterated the presence of positive selection that was predominantly evident in the MHC Class I gene. In instances of positive selection, novel mutations provide a selective advantage over different forms of mutation, and fixed mutations are predominantly adaptable even if their presence is mostly deleterious or neutral in nature [35,36]. Evidence of balancing selection has been reported as the vessel for genetic variation maintenance among these genes [37,38,39,40]. Balancing selection occurs when a set of selective processes allows for various versions of a gene to be maintained within a population, favoring differentiation in allelic make-up known as heterozygotes [40]. The results from the Tajima’s neutrality test (MEGA X) showed a total number of 38 segregating sites for the hatchery population at the MHC Class I locus, where total nucleotide diversity was 0.037, with a Tajima test statistic of 0.145 [41]. The wild population had 41 segregating sites with a nucleotide diversity of 0.046, and a Tajima test statistic of 1.28. A Tajima test statistic of 0 means that observed heterozygosity and expected heterozygosity are equal (He = Ho), whereas a test statistic greater than zero reflects higher than expected heterozygosity likely resulting from a scarcity of rare alleles [41]. This could result from population contractions and aligns with balancing selection as the driving selective force. Despite an elevated Tajima test statistic for the MHC Class I among the wild salmon, these results were not statistically significant under a 95% confidence limit. The codon-based test for neutrality analysis method in MEGA (version X; 500 bootstrap replicates) was used to determine the probability of rejecting the null hypothesis of strict neutrality when averaging overall sequence pairs among Class I MHC alleles [42]. The *p*-value of this test was marginally significant for the wild population at the MHC Class I loci (*p* = 0.06) but not significant for the hatchery population (*p* = 0.14). For the Class II loci, the hatchery population had an overall nucleotide diversity of 0.004, with only four segregating amino acid sites. Tajima’s D was −1.17. Wild populations had a nucleotide diversity of 0.002 with three segregating sites. The Tajima test statistic for this population was −1.48. In instances of a negative Tajima’s D, heterozygosity is lower than would be expected (He > Ho). This means that there is an abundance of rare alleles present within a population and may be reflective of population expansion after a genetic bottleneck, or a selective sweep of a beneficial allele resulting in it being fixed within a population (i.e., positive selection) [41]. The Nei and Gojobori test of neutrality resulted in a probability of (*p*) = 0.09 for rejecting the null hypothesis of strict neutrality (dN = dS) among hatchery individuals [43]. The wild stock had a *p*-value of (*p*) 0.13 for the Nei–Gojobori test of neutrality.

The prevalence of mutation was considerably higher in regions of the PBR comparative to that of non-coding regions. Sites of positive selection were predominantly found at loci related to the PBR, suggesting that amino acids of the peptide binding region are undergoing selection that favors diversity for Class I and II loci. In theory, the more polymorphic these sites are, the greater the defense against parasites and pathogens the individual, its offspring, and the overall population should have. Previous studies echo these findings [2] and go on to suggest the presence of recombination within the MHC Class I loci of several British Columbian populations. Recombination is a process by which pieces of gametic DNA from mating pairs are split and recombined in a manner that leads to a novel combinations of alleles [44]. The contribution of recombination as a vector for high levels of MHC diversity is hotly debated, but this presents an opportunity for further understanding the processes driving MHC diversity. The presence of recombinant genes would allow for multiple point mutations to occur per recombination event as opposed to individual site mutations, allowing for expanded adaptability at PBR sites [45]. Tests for recombination in unison with assessments of MHC-driven mate selection would give a more holistic view of the factors driving the highly variable nature of the MHC.

There are many vertebrate species that utilize olfactory cues as a method of identifying mates with dissimilar MHC genes, which is identifiable through scent [46]. The development of two hypotheses (the “good genes” hypothesis and the “compatibility” hypothesis) predict additive (good genes) or non-additive (compatibility) fitness benefits bestowed upon offspring suggests several ways in which MHC-driven mating preference can increase the survival, growth rate, disease resistance, and body size of offspring [32,46,47]. In a study conducted on the freshwater Chinese Rose bitterling fish (*Rhodeus ocellatus*), it was determined that female mate preference coincided with the genetic compatibility of the male [48]. This genetic compatibility was determined to be directly correlated with MHC dissimilarity [47]. Thus, it was determined that genetic compatibility is the vessel in which the benefit from mate choice is determined and that vessel is driven by olfactory cues signaling MHC dissimilarity [47]. A study on Atlantic salmon revealed that MHC-mediated mate choice and natural selection (parasite-mediated) were the driving factors maintaining the MHC diversity among offspring [49]. The offspring of the hatchery-reared salmon were found to have considerably higher parasite loads and were four times more susceptible to infection when compared to free-mating salmon [49]. The hatchery-reared salmon displayed similar levels of MHC diversity, but the wild salmon offspring showed higher levels of MHC dissimilarity [49]. As is the case with hatchery bred Chinook salmon, the selection of mating partners is not part of the equation.

Several studies have suggested that salmonid females mate non-randomly at MHC loci, preferring mates that offer greater genetic variability at these loci, but randomly on the basis of relatedness through post-copulatory selection [44,50]. Considering that effective population sizes of salmon are already relatively small, it would make total inbreeding avoidance rather difficult. Based on this information, one of the hypotheses of the present study was that samples taken from the Deshka River would display greater levels of observed heterozygosity (Ho) relative to expected heterozygosity (He), suggesting that individuals with similar phenotypes mate with one another less frequently than would be expected under random mating patterns, meaning that mating has not occurred due solely to chance. Conversely, since spawning in the William Jack Hernandez Sport Fish hatchery is managed directly by hatchery personnel, mate choice is not expected to play as significant an ecological role as it would in wild populations. Consequently, we would not expect to see this discrepancy (He = Ho) in hatchery fish tested in this study. The alternative hypothesis of this study posits that post-mating selection is driving higher levels of observed (Ho > He) heterozygosity as opposed to mate selection among hatchery salmon.

Both populations exhibited an excess of heterozygotes for the MHC Class I and II loci suggesting selection favoring heterozygous individuals at the MHC. This also lends support to the alternative hypothesis that post-mating selection is driving high levels of heterozygosity among hatchery and wild fish. This suggests that Chinook salmon target mates based on complimentary MHC genes to increase the fitness of their offspring, particularly at the PBR of MHC genes. The information relayed in [32] suggests that this is not the result of genome-wide inbreeding avoidance (which would be difficult given smaller population sizes of subpopulations) but specifically targets the genotypic complexity of amino acids within the PBR. The low level of expected heterozygosity is likely the result of expected levels of inbreeding given both populations are isolated from one another, and statistical analyses used relatively smaller samples sizes (96 individuals per treatment). Higher than expected levels of observed heterozygosity suggest gene flow from a previously isolated population containing a unique set of alleles. Levels of heterozygosity were higher for the hatchery population at both loci, nucleotide diversity was considerably lower, and fewer alleles were present. Fixation index values were low for both populations, which indicates a lack of diversity between populations, which is to be expected for communities of the same species that are geographically adjacent to one another. The study by Evans et al. [2] discussed the heterozygote advantage found among Chinook salmon juveniles with high levels of nucleotide diversity at the Class I loci but conversely found that individuals with low nucleotide at the Class II loci had lower juvenile mortality rates. The findings of the tests for heterozygosity further the narrative that the MHC loci are under balancing selection.

Based on the findings of the statistical analyses discussed above, it can be determined that while high levels of non-synonymous substitutions are occurring at PBR codons in both genes, they remain under balancing selection. Measures of dN/dS revealed a highly polymorphic MHC Class I gene, but based on Tajima’s D statistic, it is not likely that this gene is evolving in a manner that rejects neutrality. However, it would appear that the benefit of heterogeneity at the PBR is pushing the expanded allelic diversity at this locus. Having a high dN/dS value at the PBR codons suggests that this region is undergoing selection that favors amino acid diversity resulting in an advantageous sweep of genetic variants. This suggests that the Chinook salmon studied here should be able to mount a substantial immune response to a variety of endogenous pathogens. The CD8+ T cells produced by the MHC class I genes serve as mediators of adaptive immune systems through immunosurveillance that result in the elimination of tumor cells, virally infected cells, and intracellular pathogens [51]. The Class II B1 loci play a key role in the eradication of microbes, cytotoxins, and various antigens through the immune response after MHC Class II interaction with exogenous pathogens and active CD4+ T cells [52,53]. CD4+ T cells also aid in the suppression of the immune response based on the identification of antigen-specific cytokines [53]. While the Class II PBR codons did not show rates of non-synonymous substitutions on par with the Class I sites, they did show higher overall substitution rates at non-binding codons, specifically those adjacent to the PBR. While balancing selection appears to be the driving selective force for both loci, it appears as though heterozygotes at PBR sites are selectively favorable based on pathogenic response. Furthermore, the low level of allelic diversity among the Class II loci could reflect lesser pathogenic pressure comparatively among salmonid species in the northern portion of their range. This could mean that certain advantageous alleles are more fixed within a population that deals with a lightened pathogenic load compared to other spatially different studies. The Chinook salmon in the more southern portions of their range are likely subject to a greater number of pathogens due to introgression of less genetically fit individuals as well as climate pressures resulting from warming oceans.

## 5. Conclusions

Using a medley of statistical analyses, this study demonstrated high genetic variability is being preserved among MHC Class I and II genes and highlighted the selective pressures that are driving such high levels of variation. Levels of dN/dS showed high rates of non-synonymous substitutions at the PBR codons found in MHC genes but not at overall levels that would reject neutral evolutionary theory. In other words, the changes occurring at these genes are not likely to be causing any detrimental biological changes in the salmon in relation to immune health. The salmon originating from the William Jack Hernandez Hatchery show similar levels polymorphism to those of the wild stock from the Deshka River at both MHC loci. This bodes well for the hatchery practices utilized by the state of Alaska and shows the importance of championing genetic composition throughout hatchery applications but only focuses on one piece of the puzzle. Although the MHC genes studied here have maintained healthy levels of diversity, this study does not encompass the entire genomic health of the fish.

As such it invites a genome-wide study such as mapping Single Nucleotide Ploymophisms (SNPs) that could further our understanding of the role of MHC haplotypes in mate choice while also providing new biomarkers for optimizing genetic diversity and adaptive resiliency of fish produced by aquaculture programs targeting Chinook salmon. 

## Figures and Tables

**Table 1:** MHC Class I and II locus-specific oligo sequences and PCR reaction profiles used in the present study following optimized protocols described in references [4,15].

Exon	Sense	Name	bp	Primer	PCR Profile-35 Cycles
Class I					
A1	Sense	A1RA	260–266	5′-TGACTCACGCCCTGAAGTA-3′	94 C (30 s) 50 C (1 min) 72 C (1 min)
	Anti	A1FA		5′-CTCCACTTTGGTTAAAACG-3′	
A2–A3	Sense	A2RUA	750	5′-GTTAACCAGTGGATG[CA][AT]TGG[AC]TGTGGAG-3′	94 C (30 s) 55 C (1 min) 72 C (3 min)
	Anti	A3FUA		5-′GAGTTGAACCACACA[CG]T[CG]ATA-3′	
Class II					
B1	Sense	B1RA	257	5′-CCGATACTCCTCAAAGGACCTGCA-3′	94 C (30 s) 55 C (1 min) 72 C (1 min)
	Anti	B1FA		5′-GGTCTTGACTTG[AC]TCAGTCA-3′	

**Table 2:** AMOVA for variation in MHC Class I A1 and MHC Class II B1 alleles.

AMOVA CLASS I A1				
Source of Variation	d.f.	Sum of Squares	Variance Components	% Variation
Among Populations	4	4.369	0.019 VA	4
Within Populations	200	91.573	0.458 VB	96
Total	204	95.941	0.477	100
**AMOVA Class II B2**				
Among Populations	1	0.599	0.00525 VA	4.87
Within Populations	194	19.865	0.10240 VB	95.13
Total	195	20.464	0.10764	100

VA: variance component A; VB: variance component B.

**Table 3:** Observed vs. expected heterozygosity: Class I A1: N = number of individuals per population; Ho = observed heterozygosity; He = expected heterozygosity; Polymorphic loci = number of loci for which Hardy–Weinberg Equilibrium was rejected.

Population		N	Ho	He		Polymorphic Loci (Rejects HWE)	Nucleotide Diversity
Hatchery	Mean	34	0.898	0.478	Mean	38	0.037
	SD		0.269	0.130	SE		0.020
Wild	Mean	86	0.879	0.468	Mean	41	0.046
	SD		0.287	0.137	SE		−0.023
Total	Mean	205	0.838	0.450	Mean	147	0.060
	SE		0.333	0.167	SE		

**Table 4:** Observed vs. expected heterozygosity MHC Class II B1: N = number of individuals per population; Ho = observed heterozygosity; He = expected heterozygosity; Na = number of alleles per loci; Ne = number of effective alleles; I = Shannon’s index; uHe = unbiased expected heterozygosity.

Population		N	Na	Ne	I	Ho	He	uHe
Hatchery	Mean	83.000	1.845	1.819	0.569	0.817	0.409	0.412
	SE	0.000	0.029	0.026	0.018	0.026	0.013	0.013
Wild	Mean	60.000	1.826	1.818	0.567	0.817	0.409	0.412
	SE	0.000	0.027	0.026	0.018	0.026	0.013	0.013

**Table 5:** Codon-specific dN/dS.

	Codon	dN/dS	Codon	dN/dS
PBR	27	2.939	22	1.741
Non-PBR	49	0.908	51	1.048
Total	76	1.732	73	1.115

PBR: Protein Binding Region.

## Data Availability

All original aligned DNA sequences analyzed for this study were deposited into the NIH NCBI Nucleotide Database (https://www.ncbi.nlm.nih.gov/nucleotide/) (accessed on 6 February 2023) under GenBank Accession Nos. OQ091972-OQ092097 (MHC class I-α1 locus) and OQ092098-OQ092242 (MHC class II-β1 locus).

## Supplementary Materials

The following supporting information can be downloaded at: https://www.mdpi.com/article/10.3390/ani13040593/s1, Figure S1: MHC Class I A1 PCR product bands in raw gel image (228 bp) [left panel]; MHC Class II B1 product bands (213 bp) [right panel]; Table S1: *O. tshawytscha* Class I A1 alleles indicating polymorphic sites aligned with the published consensus sequence and NCBI GenBank outgroup data for *Salmo salar*, *Oncorhynchus mykiss* and *Onchorhynchus gorbuscha* [4,15]; Table S2: *O. tshawytscha* Class II B1 alleles indicating polymorphic sites aligned with the published consensus sequence from [2,4].
