# Supplementary material for: Evaluating the Potential Fitness Effects of Chinook Salmon (Oncorhynchus tshawytscha) Aquaculture Using Non-Invasive Population Genomic Analyses of MHC Nucleotide Substitution Spectra
Source: Animals (Basel). 2023 Feb 8;13(4):593. doi: 10.3390/ani13040593 (PMC9951711; doi:10.3390/ani13040593)
Supplement: Supplementary file 1 [file animals-13-00593-s001.zip › animals-2142632 Supplementary.pdf]

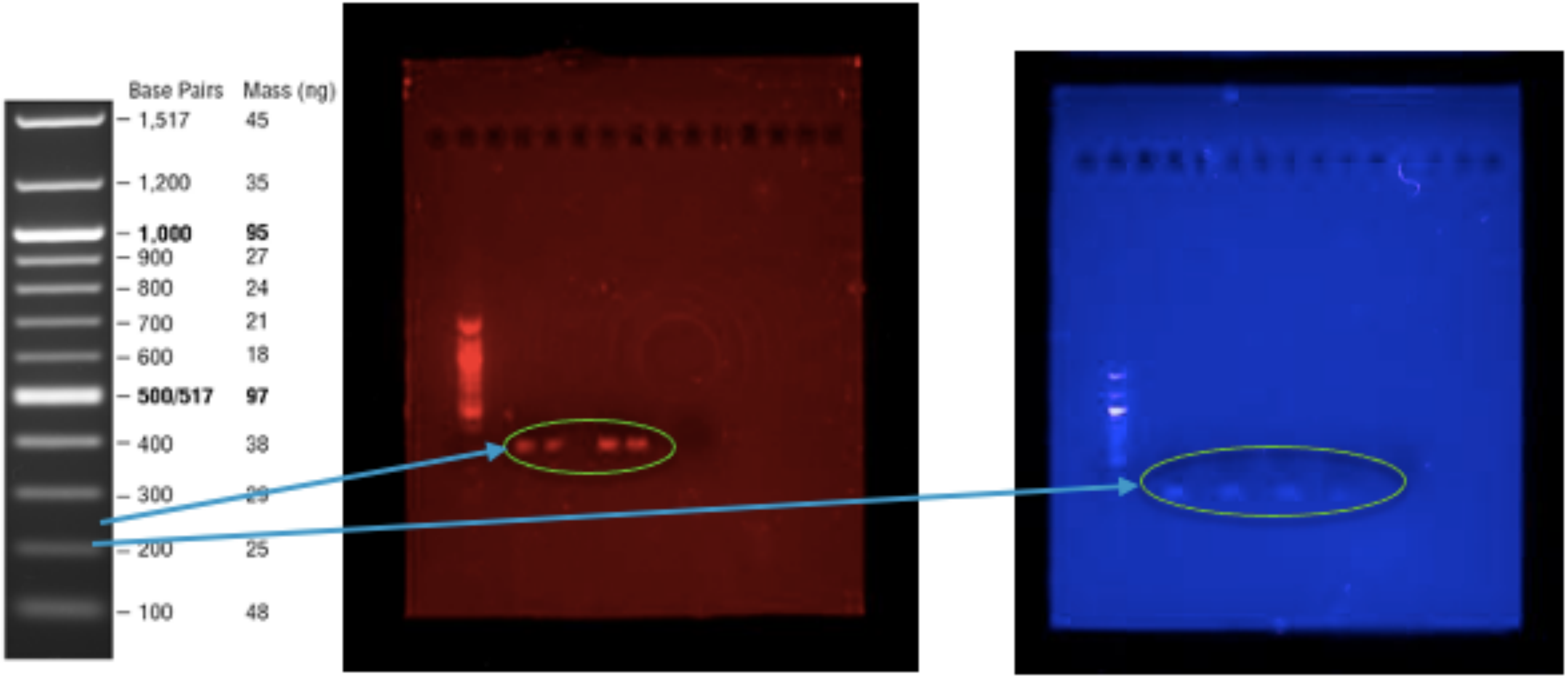

Supplementary Figure S1. MHC Class I A1 PCR product bands in raw gel image (228bp) [left panel]; MHC Class II B1 product bands (213bp) [right panel].

Supplementary Table S1. *O. tshawytscha* Class I A1 alleles indicating polymorphic sites aligned with the published consensus sequence and NCBI GenBank outgroup data for *Salmo salar, Oncorhynchus mykiss* and *Onchorhynchus gorbuscha* [4,15].

Onts-A1*1	F	Y	T	A	S	S	E	V	P	N	F	P	E	F	V	V	V	G	M	V	D	G	V	Q	M	V	H	Y	D	S	N	S	Q	R	A	V	P	K	Q	D	W	I	N	K	A	A	E	T	L	P	Q	Y	W	E	R	E	T	G	N	F	K	G	D	Q	Q	T	F	K	A	N	I	D	I	A	K	Q
HA10A1	.	.	.	.	.	.	.	.	.	.	.	.	.	.	.	I	.	.	.	.	.	.	.	.	.	.	.	.	.	.	.	.	.	.	.	.	.	.	.	.	.	V	.	.	.	.	D	-	-	.	.	.	.	.	.	N	.	.	.	G	.	.	A	.	.	.	.	.	.	.	.	.	.	V	.	.
HA12A1	.	.	.	.	.	.	.	.	.	.	.	.	.	.	.	I	.	.	.	.	.	.	.	.	.	.	.	.	.	C	.	.	.	.	.	.	.	.	.	.	.	.	.	.	.	.	D	-	-	.	.	.	.	.	.	N	.	.	.	G	N	.	A	.	P	.	.	.	.	K	.	.	.	V	.	.
HA8A1	.	.	.	.	.	.	.	.	.	.	.	.	.	.	.	I	.	.	.	.	.	.	.	.	.	.	.	.	.	.	.	.	.	.	.	.	.	.	.	.	.	M	.	.	.	.	D	-	-	.	.	.	.	.	.	N	R	N	.	G	N	.	A	.	H	.	.	.	.	.	.	N	.	V	.	.
HB10A1	.	.	.	.	.	.	.	.	.	.	.	.	.	.	.	.	.	.	.	.	.	.	.	.	.	.	.	.	.	.	.	.	.	.	.	.	.	.	.	.	.	M	.	.	.	.	.	.	.	.	.	.	.	.	.	N	.	.	.	C	.	.	.	.	.	.	.	.	.	.	.	.	.	.	.	.
HB11A1	.	.	.	.	.	.	.	.	.	.	.	.	.	.	.	.	.	.	T	.	.	.	.	.	.	.	.	.	.	C	.	.	.	.	.	.	.	.	.	.	.	V	.	.	.	.	.	.	.	.	.	.	.	.	.	K	.	.	.	C	.	.	T	.	.	.	.	.	.	.	.	.	.	V	.	.
HD12A1	.	.	.	.	.	.	.	.	.	.	.	.	.	.	.	.	.	.	T	.	.	.	.	.	.	.	.	.	.	.	.	.	.	.	.	.	.	.	.	.	.	V	.	.	.	.	.	.	.	.	.	.	.	.	.	N	.	.	.	C	.	.	T	.	.	.	.	.	.	.	.	.	.	V	.	.
ONCORHYNCHUSMYKISS	.	.	.	.	.	.	.	.	.	.	.	.	.	.	.	.	.	.	.	.	.	.	.	.	.	.	.	.	.	.	.	.	.	.	.	.	.	.	.	.	.	M	.	.	.	.	.	.	-	-	.	.	.	.	.	.	.	.	.	C	.	.	T	.	.	.	.	.	.	.	.	.	.	V	.	.
oncorhynchusgorbuscha	.	F	.	T	.	.	.	.	.	.	.	.	.	.	.	.	.	.	.	.	.	.	.	.	.	.	.	.	.	.	.	.	.	.	.	.	.	.	.	.	.	M	.	.	.	.	.	.	-	-	.	.	.	.	.	.	.	.	I	C	.	.	T	.	.	.	.	.	.	.	.	.	.	L	.	.
salmosalar	.	.	.	.	.	.	.	.	.	.	.	.	.	.	M	.	.	.	.	.	.	.	.	.	.	.	.	.	.	.	.	.	.	.	M	.	.	.	.	.	.	M	.	.	Q	T	D	-	-	A	E	.	.	.	.	.	.	.	I	A	F	D	S	.	.	V	.	.	D	D	V	N	.	L	.	.
wa5r1	.	.	.	.	.	.	.	.	.	.	.	.	.	.	.	.	.	.	.	.	.	.	.	.	.	.	.	.	.	.	.	.	.	.	.	.	.	.	.	.	.	M	.	.	.	.	.	.	.	.	.	.	.	.	.	.	.	.	.	C	.	.	.	.	.	.	.	.	.	.	.	.	.	.	.	.
wa7a1	.	.	.	.	.	.	.	.	.	.	.	.	.	.	.	I	.	.	.	.	.	.	.	.	.	.	.	.	.	.	.	.	.	.	.	.	.	.	.	.	.	V	.	.	.	.	D	-	-	.	.	.	.	.	.	N	.	.	.	G	.	.	A	.	.	.	.	.	.	.	.	.	.	V	.	.
wa8a1	.	.	.	.	.	.	.	.	.	.	.	.	.	.	.	.	.	.	.	.	.	.	.	.	.	.	.	.	.	.	.	.	.	.	.	.	.	.	.	.	.	M	.	.	.	.	.	.	.	.	.	.	.	.	.	K	.	.	.	.	.	.	A	.	.	.	.	.	.	.	.	.	.	.	.	.
wa9a1	.	.	.	.	.	.	.	.	.	.	.	.	.	.	.	.	.	.	.	.	.	.	.	.	.	.	.	.	.	.	.	.	.	.	.	.	.	.	.	.	.	M	.	.	.	.	.	.	.	.	.	.	.	.	.	.	.	.	.	C	.	.	A	.	.	.	.	.	.	.	.	.	.	.	.	.
wb4r	.	.	.	.	.	.	.	.	.	.	.	.	.	.	.	.	.	.	.	.	.	.	.	.	.	.	.	.	.	.	.	.	.	.	.	.	.	.	.	.	.	M	.	.	.	T	.	.	.	.	.	.	.	.	.	.	.	.	.	C	.	.	A	.	.	.	.	.	.	.	.	.	.	.	.	.
wb6r	.	.	.	.	.	.	.	.	.	.	.	.	.	.	.	I	.	.	.	.	.	.	.	.	.	.	.	.	.	.	.	.	.	.	.	.	.	.	.	.	.	V	.	.	.	.	D	-	-	.	.	.	.	.	.	N	.	.	.	G	.	.	A	.	.	.	.	.	.	.	.	.	.	V	.	.
wb9a1	.	.	.	.	.	.	.	.	.	.	.	.	.	.	.	.	.	.	.	.	.	.	.	.	.	.	.	.	.	.	.	.	.	.	.	.	.	.	.	.	.	M	.	.	.	.	.	.	.	.	.	.	.	.	.	.	.	.	.	.	.	.	.	.	.	.	.	.	.	.	.	.	.	.	.	.
wc11a1	.	.	.	.	.	.	.	.	.	.	.	.	.	.	.	.	.	.	.	.	.	.	.	.	.	.	.	.	.	.	.	.	.	.	.	.	.	.	.	.	.	V	.	.	.	.	.	.	.	.	.	.	.	.	.	N	.	.	.	C	.	.	T	.	.	.	.	.	.	.	.	.	.	V	.	.
we10a1	.	.	.	.	.	.	.	.	.	.	.	.	.	.	.	.	.	.	.	.	.	.	.	.	.	.	.	.	.	.	.	.	.	.	.	.	.	.	.	.	.	V	.	.	.	.	.	.	.	.	.	.	.	.	.	.	.	.	.	C	.	.	T	.	.	.	.	.	.	.	.	.	.	.	.	.
we12a1	.	.	.	.	.	.	.	.	.	.	.	.	.	.	.	I	.	.	.	.	.	.	.	.	.	.	.	.	.	.	.	.	.	.	.	.	.	.	.	.	.	V	.	.	.	.	D	-	-	.	.	.	.	.	.	N	.	.	.	G	.	.	A	.	.	.	.	.	.	.	.	.	.	V	.	.
we8a1	.	.	.	.	.	.	.	.	.	.	.	.	.	.	.	I	.	.	.	.	.	.	.	.	.	.	.	.	.	.	.	.	.	.	.	.	.	.	.	.	.	V	.	.	.	.	D	-	-	.	.	.	.	.	.	N	.	.	.	G	.	.	A	.	.	.	.	.	.	.	.	.	.	V	.	.
wh1r	.	.	.	.	.	.	.	.	.	.	.	.	.	.	.	I	.	.	T	.	.	.	.	.	.	.	.	.	.	.	.	.	.	.	.	.	.	.	.	.	.	V	.	.	.	.	D	-	-	.	.	.	.	D	W	N	.	E	.	G	N	.	A	.	T	.	.	T	.	.	.	N	.	V	.	.
wh4r	.	.	.	.	.	.	.	.	.	.	.	.	.	.	.	I	.	.	T	.	.	.	.	.	.	.	.	.	.	.	.	.	.	.	.	.	.	.	.	.	.	V	.	.	.	.	D	-	-	.	.	.	.	D	W	N	.	D	T	G	N	.	A	.	P	.	.	T	.	.	.	N	.	V	.	.
we3r	.	.	.	.	.	.	.	.	.	.	.	.	.	.	.	.	.	.	.	.	.	.	.	.	.	.	.	.	.	.	.	.	.	.	.	.	.	.	.	.	.	V	.	.	.	.	D	-	-	.	.	.	.	D	W	N	S	N	T	G	N	.	A	.	T	P	.	T	.	.	V	N	.	V	.	.
HH9A1	.	.	.	.	.	.	.	.	.	.	.	.	.	.	.	I	.	.	.	.	.	.	.	.	.	.	.	.	.	.	.	.	.	.	.	.	.	.	.	.	.	.	.	.	.	.	D	-	-	.	.	.	.	D	W	N	R	.	T	G	N	.	A	.	A	.	.	T	.	.	V	N	.	V	.	.
wc5r	.	.	.	.	.	.	.	.	.	.	.	.	.	.	.	I	.	.	.	.	.	.	.	.	.	.	.	.	.	.	.	.	.	.	.	.	.	.	.	.	.	V	.	.	.	.	D	-	-	.	.	.	.	D	W	N	R	V	T	G	N	.	A	.	A	.	.	T	.	.	.	N	.	V	.	.
wc6r	.	.	.	.	.	.	.	.	.	.	.	.	.	.	.	I	.	.	.	.	.	.	.	.	.	.	.	.	.	.	.	.	.	.	.	.	.	.	.	.	.	V	.	.	.	.	D	-	-	.	.	.	.	D	W	N	.	E	.	G	N	V	A	.	D	P	.	T	.	.	.	N	.	V	.	.
wh6r	.	.	.	.	.	.	.	.	.	.	.	.	.	.	.	I	.	.	.	.	.	.	.	.	.	.	.	.	.	.	.	.	.	.	.	.	.	.	.	.	.	V	.	.	.	.	D	-	-	.	.	.	.	D	W	N	.	.	T	G	N	.	A	.	P	.	.	T	.	.	.	N	.	V	.	.
we4r	.	.	.	.	.	.	.	.	.	.	.	.	.	.	.	I	.	.	.	.	.	.	.	.	.	.	.	.	.	.	.	.	.	.	.	.	.	.	.	.	.	M	.	.	.	.	D	-	-	.	.	.	.	D	W	N	.	.	T	G	N	.	A	.	A	.	.	T	.	.	.	N	.	V	.	.
wd3r	.	.	.	.	.	.	.	.	.	.	.	.	.	.	.	I	.	.	.	.	.	.	.	.	.	.	.	.	.	.	.	.	.	.	.	.	.	.	.	.	.	V	.	.	.	.	D	-	-	.	.	.	.	D	W	N	.	.	T	G	N	.	A	.	A	.	.	T	.	.	.	N	.	V	.	.
wc10a1	.	.	.	.	.	.	.	.	.	.	.	.	.	.	.	.	.	.	.	.	.	.	.	.	.	.	.	.	.	.	.	.	.	.	.	.	.	.	.	.	.	V	.	.	.	.	D	-	-	.	.	.	.	.	.	K	R	V	.	G	N	.	A	.	A	.	.	T	.	.	.	N	.	V	.	.
wg12a1	.	.	.	.	.	.	.	.	.	.	.	.	.	.	.	I	.	.	.	.	.	.	.	.	.	.	.	.	.	.	.	.	.	.	.	.	.	.	.	.	.	V	.	.	.	.	D	-	-	.	.	.	.	.	.	N	S	E	.	G	N	.	A	.	D	.	.	T	.	.	V	N	.	V	.	.
wb8a1	.	.	.	.	.	.	.	.	.	.	.	.	.	.	.	.	.	.	.	.	.	.	.	.	.	.	.	.	.	.	.	.	.	.	.	.	.	.	.	.	.	V	.	.	.	.	D	-	-	.	.	.	.	.	.	N	R	V	.	G	N	.	A	.	A	.	.	T	.	.	.	N	.	V	.	.
wb2r	.	.	.	.	.	.	.	.	.	.	.	.	.	.	.	I	.	.	.	.	.	.	.	.	.	.	.	.	.	.	.	.	.	.	.	.	.	.	.	.	.	V	.	.	.	T	D	-	-	.	.	.	.	.	.	N	.	E	.	G	I	.	A	.	D	.	.	T	.	.	V	N	.	V	.	.
wb7a1	.	.	.	.	.	.	.	.	.	.	.	.	.	.	.	I	.	.	.	.	.	.	.	.	.	.	.	.	.	.	.	.	.	.	.	.	.	.	.	.	.	V	.	.	.	.	D	-	-	.	.	.	.	.	.	N	R	K	.	G	N	.	A	.	D	.	.	T	.	.	.	N	.	V	.	.
wc7a1	.	.	.	.	.	.	.	.	.	.	.	.	.	.	.	I	.	.	T	.	.	.	.	.	.	F	.	.	.	.	.	.	.	.	.	.	.	.	.	.	.	V	.	.	.	.	D	-	-	.	.	.	.	.	.	N	R	N	T	G	N	.	A	.	T	P	.	T	.	K	V	N	.	V	.	.
wd8a1	.	.	.	.	.	.	.	.	.	.	.	.	.	.	.	I	.	.	.	.	.	.	.	.	.	.	.	.	.	.	.	.	.	.	.	.	.	.	.	.	.	V	.	.	.	.	D	-	-	.	.	.	.	.	.	N	R	N	.	G	N	.	A	.	D	.	.	T	.	.	.	.	.	V	.	.
wd4r	.	.	.	.	.	.	.	.	.	.	.	.	.	.	.	I	.	.	T	.	.	.	.	.	.	.	.	.	.	.	.	.	.	.	.	.	.	.	.	.	.	V	.	.	.	.	.	-	-	.	.	.	.	.	.	N	.	D	T	G	N	.	A	.	T	P	.	T	.	.	.	N	.	V	.	.

Supplementary Table S2. *O. tshawytscha* Class II B1 alleles indicating polymorphic sites aligned with the published consensus sequence from [2, 4].

Site	1	2	3	4	5	6	7	8	9	10	11	12	13	14	15	16	17	18	19	20	21	22	23	24	25
EVANS SEQUENCE 24	G	I	E	F	I	D	S	Y	V	F	N	K	V	E	H	I	R	F	N	S	T	V	G	R	Y
HD9B1 b1r	.	.	.	.	.	.	.	.	.	.	.	.	.	.	.	.	.	.	.	.	.	.	.	.	.
HF8B1 b1r	.	.	.	.	.	.	.	.	.	.	.	.	A	.	.	.	.	.	.	.	.	.	.	.	.
HE7B1 b1r	.	.	.	.	.	.	.	.	.	.	.	.	A	.	.	.	.	.	.	.	.	.	.	.	.
hh5b1 PREMIX	.	.	.	.	.	.	.	.	.	.	.	.	A	.	.	.	.	.	.	.	.	.	.	.	.
HH11B1 b1r	.	.	.	.	.	.	.	.	.	.	.	.	A	.	Y	.	.	.	.	.	.	.	.	.	.
wa8b1 PREMIX	.	.	.	.	.	.	.	.	.	.	.	.	A	.	Y	.	.	.	.	.	.	.	.	.	.
MILLER CONSENSUS	.	.	.	.	.	.	.	.	.	.	.	.	A	.	Y	.	.	.	.	.	.	.	.	.	.

Site	26	27	28	29	30	31	32	33	34	35	36	37	38	39	40	41	42	43	44	45	46	47	48	49	50
EVANS SEQUENCE 24	V	G	Y	T	E	H	G	V	K	N	A	E	A	W	N	K	G	P	Q	L	G	Q	E	Q	A
HD9B1 b1r	.	.	.	.	.	.	.	.	.	.	.	.	.	.	.	.	.	.	.	.	.	.	.	.	.
HF8B1 b1r	.	.	.	.	.	.	.	.	.	.	.	.	.	.	.	.	.	.	.	.	.	.	.	.	.
HE7B1 b1r	.	.	.	.	.	.	.	.	.	.	.	.	.	.	.	.	.	.	.	.	.	.	.	.	.
hh5b1 PREMIX	.	.	.	.	.	.	.	.	.	.	.	.	.	.	.	.	.	.	.	.	.	.	.	.	.
HH11B1 b1r	.	.	.	.	.	.	.	.	.	.	.	.	.	.	.	.	.	.	.	.	.	.	.	.	.
wa8b1 PREMIX	.	.	.	.	.	.	.	.	.	.	.	.	.	.	.	.	.	.	.	.	.	.	.	.	.
MILLER CONSENSUS	.	.	.	.	.	L	.	.	.	.	.	.	.	.	.	.	.	.	.	.	.	.	.	.	.

Site	51	52	53	54	55	56	57	58	59	60	61	62	63	64	65	66	67	68	69	70	71	72	73
EVANS SEQUENCE 24	E	L	E	R	F	C	K	P	N	A	A	L	Q	Y	R	A	I	L	D	K	T	G	E
HD9B1 b1r	.	.	.	.	.	.	.	.	.	.	.	.	H	.	.	.	.	.	.	.	.	.	.
HF8B1 b1r	.	.	.	.	V	.	.	.	.	.	.	.	H	.	.	.	.	.	.	.	.	.	.
HE7B1 b1r	.	.	.	.	.	.	.	.	.	.	.	.	H	.	.	.	.	.	.	.	.	.	.
hh5b1 PREMIX	.	.	.	.	V	.	.	.	.	.	.	.	H	.	.	.	.	.	.	.	.	.	.
HH11B1 b1r	.	.	.	.	V	.	.	.	.	.	.	.	H	.	.	.	.	.	.	.	.	.	.
wa8b1 PREMIX	.	.	.	.	.	.	.	.	.	.	.	.	.	.	.	.	.	.	.	.	.	.	.
MILLER CONSENSUS	.	.	.	.	.	.	.	.	.	.	.	.	H	.	.	.	.	.	.	.	.	.	.
